# Visuospatial alpha and gamma oscillations scale with the severity of cognitive dysfunction in patients on the Alzheimer’s disease spectrum

**DOI:** 10.1186/s13195-021-00881-w

**Published:** 2021-08-17

**Authors:** Alex I. Wiesman, Daniel L. Murman, Pamela E. May, Mikki Schantell, Sara L. Wolfson, Craig M. Johnson, Tony W. Wilson

**Affiliations:** 1 Montreal Neurological Institute, McGill University, 845 Sherbrooke St W, Montreal, QC H3A 0G4 Canada; 2 Department of Neurological Sciences, University of Nebraska Medical Center, Omaha, NE USA; 3 Memory Disorders & Behavioral Neurology Program, UNMC, Omaha, NE USA; 4 Institute for Human Neuroscience, Boys Town National Research Hospital, Omaha, NE USA; 5 Geriatrics Medicine Clinic, UNMC, Omaha, NE USA; 6 Department of Radiology, UNMC, Omaha, NE USA

**Keywords:** Neural oscillations, Visuospatial processing, Magnetoencephalography, Source imaging, Alzheimer’s disease

## Abstract

**Background:**

Entrainment of neural oscillations in occipital cortices by external rhythmic visual stimuli has been proposed as a novel therapy for patients with Alzheimer’s disease (AD). Despite this increased interest in visual neural oscillations in AD, little is known regarding their role in AD-related cognitive impairment and in particular during visuospatial processing.

**Methods:**

We used source-imaged magnetoencephalography (MEG) and an established visuospatial processing task to elicit multi-spectral neuronal responses in 35 biomarker-confirmed patients on the AD spectrum and 20 biomarker-negative older adults. Neuronal oscillatory responses were imaged to the level of the cortex, and group classifications and neurocognitive relationships were modeled using logistic and linear regression, respectively.

**Results:**

Visuospatial neuronal oscillations in the theta, alpha, and gamma ranges significantly predicted the classification of patients on the AD spectrum. Importantly, the direction of these effects differed by response frequency, such that patients on the AD spectrum exhibited weaker alpha-frequency responses in lateral occipital regions, and stronger gamma-frequency responses in the primary visual cortex, as compared to biomarker-negative older adults. In addition, alpha and gamma, but not theta, oscillations robustly predicted cognitive status (i.e., MoCA and MMSE scores), such that patients with neural responses that deviated more from those of healthy older adults exhibited poorer cognitive performance.

**Conclusions:**

We find that the multi-spectral neural dynamics supporting visuospatial processing differentiate patients on the AD spectrum from cognitively normal, biomarker-negative older adults. Oscillations in the alpha and gamma bands also relate to cognitive status in ways that are informative for emerging clinical interventions.

## Background

Alzheimer’s disease (AD) is increasingly recognized as a clinical spectrum of pathological change, including early-stage insults to circuit-level neuronal function [1]. Although primary sensory systems are traditionally thought to be spared until late in the AD disease course, an emerging literature has suggested that rhythmic gamma-frequency visual stimulation might attenuate amyloid-β load and rescue cognitive function, by way of microglial recruitment and enhanced hemodynamic response [2–6]. This groundbreaking line of research has spawned multiple clinical trials and, more generally, has led to an increased interest in the role of rhythmic neuronal activity in AD pathology, particularly in the visual system.

Functional magnetic resonance imaging (fMRI) has been used extensively to study visual processing in patients with AD, with a notable focus on aberrant hemodynamic responsiveness during visuospatial tasks. This literature has generally reported a decrease in stimulus-related responses in the occipital cortex [7–9], but has provided no information regarding the spectral content of these pathophysiological changes. Understanding the frequency definitions of such changes is imperative, as it is well-supported that even spatially overlapping neural responses can represent divergent information-processing mechanisms, dependent on their spectral content [10, 11]. Further, previous work has indicated that the impact of clinical disorders on such neural responses is also often mediated by their spectral content [12, 13].

Visuospatial processing is known to recruit a series of stereotyped multi-spectral neural oscillatory responses in posterior cortices [12, 14–22]. These responses commonly include (1) an early theta-frequency (~ 3–7 Hz) synchronization in the primary visual cortex, important for initial alerting to salient spatial features; (2) a later parieto-occipital desynchronization in the alpha band (~ 7–14 Hz), widely supported as indexing visual dis-inhibition in a retinotopic fashion; and (3) an early gamma-frequency (~ 50–80 Hz) synchronization, which is known to facilitate the processing of stimulus features. Regarding neuronal oscillations in AD, previous research has exclusively focused on early visual processing and has found that low-frequency responses in the delta and theta range appear to be preferentially impacted, with high-frequency alpha and gamma responses left relatively unaffected [23–27]. However, studies of neural oscillatory activity during the resting-state (i.e., when no stimuli are presented) have reported disturbances in both the alpha [28, 29] and gamma [30] bands in patients with AD. Potentially accounting for this discrepancy in previous research, no studies of visual neural oscillations in AD have required participants to recruit “higher-order” visuospatial abilities, which are impacted early and considerably in the course of the disease [31, 32].

In this study, we assess the utility of neuronal oscillatory responses during visuospatial processing for the differentiation of patients on the AD spectrum from cognitively normal, biomarker-negative older adults. Towards this goal, we leverage the high-spatio-temporal resolution of source-imaged, task-based magnetoencephalography (MEG), and logistic regression modeling. To further investigate the importance of these visual neuronal responses for clinical cognitive declines in patients with AD, we relate them to general cognitive status (i.e., MoCA and MMSE scores) using a general linear approach. We expected that these multi-spectral neuronal dynamics would significantly predict the classification of patients on the AD spectrum, as well as track cognitive status in these patients and that the nature of these relationships would be highly informative in understanding visuospatial cognitive pathology in AD. Specifically, we expected lower delta/theta responses in patients on the AD spectrum, given previous reports of a similar effect during early visual processing. In contrast, as our task required the recruitment of higher-order visuospatial abilities, we hypothesized that gamma- and alpha-frequency oscillatory responses would more closely mirror the resting state literature, where decreased alpha activity and increased gamma activity have been reported [28–30]. We did not have strong hypotheses as to the direction in which these neural aberrations might relate to behavior, since it is not well known whether they are pathological or compensatory in nature.

## Methods

### Participants

Forty-four patients with amnestic mild cognitive impairment (aMCI; *N* = 21) or mild probable AD (*N* = 23), as determined by a fellowship-trained neurologist specializing in memory disorders using standard clinical criteria [33], were enrolled in this study. One participant with probable AD disenrolled from the study due to COVID-19-related safety concerns, one aMCI patient was excluded due to a major incidental finding, and four others (1 probable AD; 3 aMCI) were excluded after whole-brain positron emission tomography (PET) imaging with florbetapir ^18^F indicated amyloid-negativity. Three additional participants with probable AD were excluded due to poor performance on the MEG visuospatial task (i.e., accuracy ≤ 55% correct) or an inability to complete the task. The remaining 35 biomarker-confirmed patients on the AD spectrum were compared to a control group of twenty older adults with normal cognition (19 amyloid-negative and one without amyloid biomarkers). Group neuropsychological profiles and demographics can be found in Table 1. The groups were matched on key demographics except for age (i.e., AD group was slightly younger), which was included as a nuisance covariate in all statistical modeling. Exclusion criteria included any medical illness affecting CNS function, any neurological disorder (other than AD/aMCI), history of head trauma, moderate or severe depression (Geriatric Depression Scale ≥ 10), and current substance abuse. The Institutional Review Board at the University of Nebraska Medical Center reviewed and approved this investigation. Written informed consent was obtained from each participant (and for patients, from their informant as well) following a detailed description of the study. In cases where the capacity to consent was questionable, informed assent was obtained from the research participant, in addition to informed consent from a legally authorized representative.

**Table 1:** Participant demographics, neuropsychological profiles, and task performance

	**Age** (years)	**Sex** (% female)	**Handedness** (# left)	**Education** (years)	**Accuracy** (%)	**RT** (ms)	
**CN**	72.70 (4.73)	60	1	16.60 (2.87)	97.47 (0.04)	579.60 (70.11)	
**ADS**	69.3 (7.14)	51	2	15.57 (2.77)	94.16 (0.08)	621.79 (117.08)	
***P***	.043	.539	.911	.203	.258	.146	
	**MoCA**^**a**^	**MMSE**	**Memory**	**Learning**	**Verbal Function**	**Attention**	**Processing Speed**
**CN**	27.43 (1.99)	29.20 (1.06)	0.33 (0.56)	0.60 (0.76)	0.18 (0.76)	0.53 (0.60)	0.66 (0.83)
**ADS**	19.51 (4.71)	23.94 (4.09)	− 2.32 (0.66)	− 2.02 (0.88)	− 0.92 (0.97)	− 0.70 (1.07)	− 0.72 (1.33)
***P***	< .001	< .001	< .001	< .001	< .001	< .001	< .001

*CN* Cognitively normal, *ADS* Alzheimer’s disease spectrum, *MoCA* Montreal Cognitive Assessment, *MMSE* Mini-Mental State Exam

^a^*n* = 49

### Neuropsychological testing

After screening and informed consent, participants underwent a battery of neuropsychological tests, with raw scores for each participant being converted to demographically adjusted *z* scores based on published normative data [34–37]. This battery was developed in collaboration with a clinical neuropsychologist specializing in memory disorders and focused on five cognitive domains generally impacted in patients with AD: *verbal memory* (Wechsler Memory Scale [WMS-IV] Logical Memory II Delayed Recall and Recognition [38]; Hopkins Verbal Learning Test-Revised [HVLT-R] Delayed Recall and Recognition Discriminability Index [39]), *learning* (WMS-IV Logical Memory I Recall [38]; HVLT-R Learning Trials 1–3 [39]), *attention and executive function* (Wechsler Adult Intelligence Scale [WAIS-IV] Digit Span Forward, Backward, and Sequencing [37]; Trail Making Test Part B [35]), *language* (Boston Naming Test [35]; Controlled Oral Word Association Test/Phonemic Verbal Fluency [35]; Animals/Semantic Verbal Fluency [35]), and *processing speed* (WAIS-IV Digit Symbol Coding [37]; Trail Making Test Part A [35]). Demographically corrected *z* scores based on test-specific normative data were averaged to create composite cognitive domain *z* scores for each participant. In addition, instrumental activities of daily living (IADLs) were measured (with an informant for patients on the AD spectrum) using the Functional Activities Questionnaire (FAQ) [40], and general cognitive status was measured using the Montreal Cognitive Assessment (MoCA) [41] and the Mini-mental State Examination (MMSE) [42].

### Florbetapir ^18^F positron emission tomography

Combined PET/CT data using ^18^F-florbetapir (Amyvid™, Eli Lilly) and a GE Discovery MI digital scanner (Waukesha, WI) were collected following the standard procedures described by the Society of Nuclear Medicine and Molecular Imaging (3D acquisition; single intravenous slow-bolus < 10 mL; dose = 370 MBq; waiting period = 30–50 min; acquisition = 10 min; [43]). Images were attenuation corrected using the CT data, reconstructed in MIMNeuro (slice thickness = 2 mm; [44]), converted to voxel standardized uptake values based on body weight (SUVbw), and normalized into MNI space. Each scan was read by a fellowship-trained neuroradiologist blinded to group assignment and assessed as being “amyloid-positive” or “amyloid-negative” using established clinical criteria [44]. At this stage, patients who were amyloid-negative were excluded from the AD spectrum group. Images were then normalized to the crus of the cerebellum (SUIT template; [45]) to generate voxel-wise maps of SUV ratios (SUVR; [46]). To test for covariance between amyloid uptake and MEG metrics, SUVRs were extracted from the same voxel locations identified in the MEG analysis and averaged bilaterally (see MEG preprocessing and sensor/source-level statistics).

### Visuospatial processing experimental paradigm

**Fig. 1:**
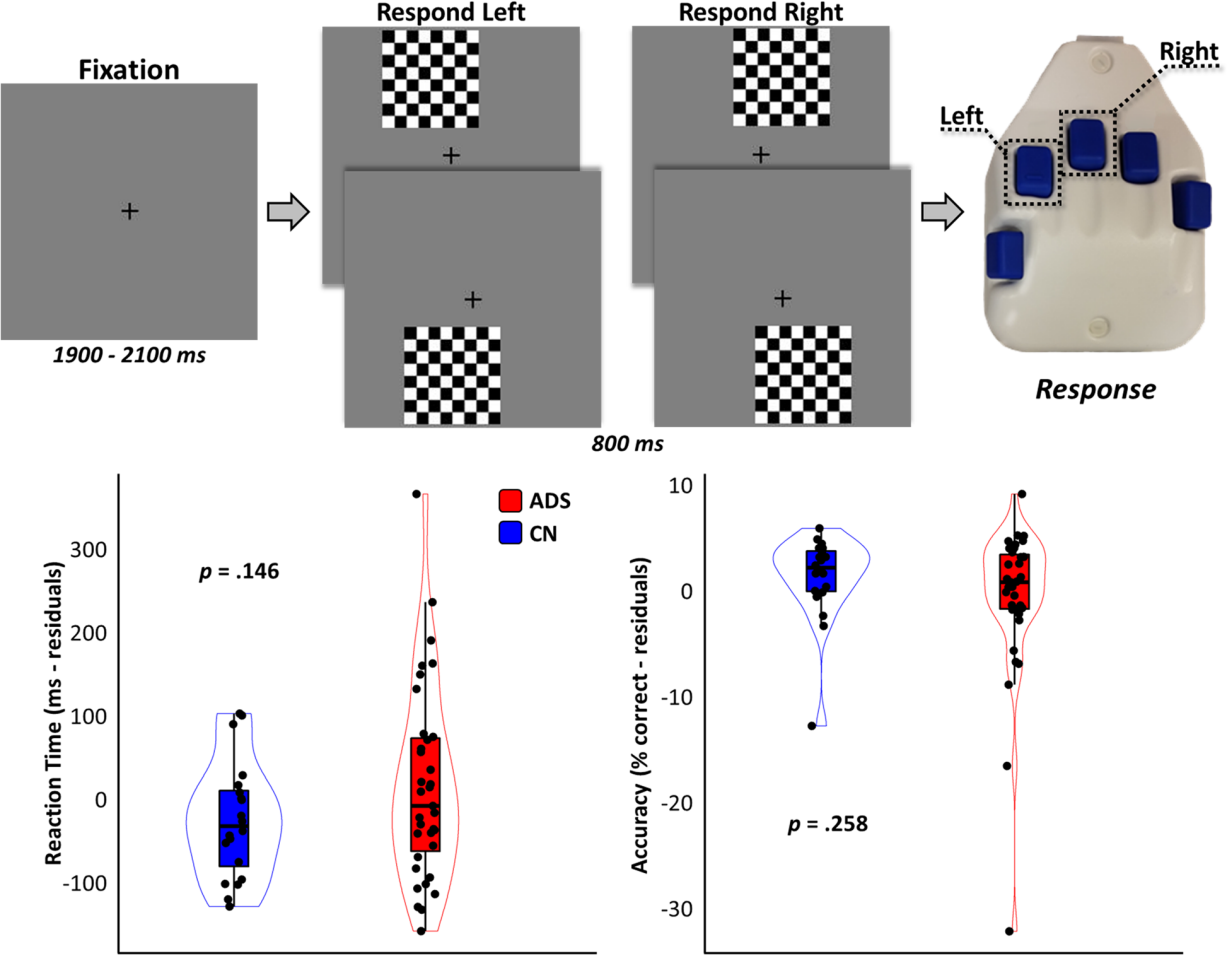
Visuospatial processing task design and behavioral performance. The visuospatial task utilized for this study is displayed above, and described in greater detail in the “Methods” section. Behavioral performance on this task is displayed per group (cognitively normal [CN]: blue; Alzheimer’s disease spectrum [ADS]: red). Box plots represent group residual means, first and third quartiles, and minima and maxima, and violin plots show the probability density

Participants completed 240 trials of a visuospatial discrimination task (Fig. 1, top), which has been extensively described and validated in previous work [12, 14–16, 20, 21, 47], concurrent with MEG recording. During this task, participants were seated in a magnetically shielded room and indicated the position of a grid by a right-handed button press (left = index finger; right = middle finger). Visual stimuli were delivered using e-Prime v2.0 (Psychology Software Tools, Pittsburgh, PA) and back-projected onto a semi-translucent non-ferromagnetic screen at an approximate distance of 1.07 m, using a Panasonic PT-D7700U-K model DLP projector with a refresh rate of 60 Hz and a contrast ratio of 4000:1.

Importantly, the relative simplicity of this task was by design. When designing a task-based neuroimaging study of patients with cognitive impairments, one typically has to choose between having these patients perform tasks that are inherently more difficult for them than for healthy adults, resulting in systematically reduced performance, or having them perform tasks that tap cognitive domains that are known to be affected by the disease but do not challenge them beyond their remaining capabilities in that domain. While both approaches have their advantages, in this case, we decided to use a task that was exceedingly simple, but still by necessity required the participants to process the visuospatial information of the stimuli. This approach has two key benefits: (1) it allows us to compare neural activity between two groups of participants that we can confidently say are both perfectly capable of performing the task at hand, ensuring their recruitment of primarily visuospatial cognitive processes and (2) it allows us to collect functional neuroimaging data from a patient group with a much wider range of cognitive abilities. In our view, point (2) is particularly important, as cognitive neuroimaging studies that employ more difficult tasks by nature either restrict their sampling to patients with relatively high cognitive functioning or forfeit the ability to show that patients were performing the task accurately. Of course, the latter severely limits interpretation, as one cannot guarantee the underlying neural responses actually support the target cognitive function.

### MEG data acquisition

Our MEG data acquisition, structural coregistration, preprocessing, and sensor-/source-level analyses closely followed the analysis pipeline of previous manuscripts [12, 48]. All recordings were conducted in a one-layer magnetically shielded room with active shielding engaged. Neuromagnetic responses were sampled continuously at 1 kHz with an acquisition bandwidth of 0.1–330 Hz using a 306-sensor Elekta/MEGIN MEG system (Helsinki, Finland) equipped with 204 planar gradiometers and 102 magnetometers. Participants were monitored during data acquisition via real-time audio–video feeds from inside the shielded room. Each MEG dataset was individually corrected for head motion and subjected to noise reduction using the signal space separation method with a temporal extension [49]. Only data from gradiometers were used for further analysis.

### Structural MRI processing and MEG coregistration

Preceding MEG measurement, four coils were attached to the participant’s head and localized, together with the three fiducial points and scalp surface, using a 3D digitizer (Fastrak 3SF0002, Polhemus Navigator Sciences, Colchester, VT, USA). Once the participant was positioned for MEG recording, an electric current with a unique frequency label (i.e., 293, 307, 314, and 321 Hz) was fed to each of the coils. This induced a measurable magnetic field and allowed each coil to be localized in reference to the sensors throughout the recording session. Since coil locations were also known in head coordinates, all MEG measurements could be transformed into a common coordinate system. With this coordinate system, each participant’s MEG data were co-registered with their own structural T1-weighted MRI data using BESA MRI (Version 2.0) prior to source-space analysis. Structural MRI data were aligned parallel to the anterior and posterior commissures and transformed into standardized space. Following source analysis (i.e., beamforming), each participant’s 4.0 × 4.0 × 4.0 mm functional images were also transformed into standardized space using the transform that was previously applied to the structural MRI volume and spatially resampled.

### MEG preprocessing and sensor/source-level statistics

Cardiac and blink artifacts were removed from the data using signal-space projection (SSP), which was subsequently accounted for during source reconstruction [50]. The continuous magnetic time series was then filtered between 0.5 and 200 Hz plus a 60-Hz notch filter and divided into 2700 ms epochs, with the baseline extending from − 400 to 0 ms prior to the onset of the visual stimulus. At this point, only trials with correct responses were considered for further analysis. Epochs containing artifacts were rejected using a fixed threshold method, supplemented with visual inspection (mean amplitude threshold: 1065.18 [SD = 240.98] fT/cm; mean gradient threshold: 205.50 [SD = 100.09] fT/(cm*ms)). An average of 201.86 (SD = 23.78) trials was used for further analysis. Importantly, none of our statistical comparisons were compromised by significant group differences in trial number nor artifact thresholds (Mann–Whitney *U* test; trial number: *p* = 0.234; amplitude threshold: *p* = 0.588; gradient threshold: *p* = 0.186).

We next transformed the post-artifact-rejection epochs into the time–frequency domain using complex demodulation [51, 52]. The time–frequency analysis was performed with a frequency-step of 2 Hz and a time-step of 25 ms between 4 and 100 Hz, using a 4 Hz lowpass finite impulse response (FIR) filter with a full-width half maximum (FWHM) in the time domain of ~ 115 ms. The resulting spectral power estimations per sensor were averaged over trials to generate time–frequency plots of mean spectral density, which were normalized by the baseline power of each respective bin ((active-baseline)/baseline), calculated as the mean power during the − 400 to 0 ms time period. The time–frequency windows used for the source analysis were determined by means of a paired-sample cluster-based permutation test against baseline across all participants and the entire frequency range (4–100 Hz), with an initial cluster threshold of *p* < 0.001 and 10,000 permutations.

Time–frequency resolved beamformer source images were computed using the dynamic imaging of coherent sources approach (DICS; [53]), which uses the time–frequency averaged cross-spectral density to calculate voxel-wise estimates of neural power and/or coherence. Following convention, we computed noise-normalized, source power per voxel in each participant using active (i.e., task) and passive (i.e., baseline) periods of equal duration and bandwidth. This approach generated three-dimensional participant-level pseudo-t maps per each time–frequency cluster identified in the sensor-level analysis. These voxel-wise maps of oscillatory neuronal response amplitude were averaged both within- and across-groups for display purposes, and the voxel of maximum amplitude (i.e., the peak voxel in the occipital cortex) in each hemisphere per oscillatory response was identified in the grand-averaged map. For enhanced visualization of the nature of these responses, virtual sensor data were extracted from these grand-average occipital peak voxels and decomposed into time–frequency space to derive the amplitude envelope of the neural signal. To test hypothesized classification effects and relationships to cognition, beamformer neural response amplitude values were extracted from the same peak voxels per participant and oscillatory response and averaged bilaterally across the hemispheres.

### Statistical analyses and software

To test the utility of these oscillatory occipital responses for classifying patients on the AD spectrum, we computed a logistic regression model with the following form: group ~ θ + α + γ + age. General linear models were used to test for hypothesized covariance between these neural responses and cognitive function (i.e., MoCA and MMSE scores) with the following form: cognitive scores ~ θ + α + γ + group + age. In addition, to test for linear relationships between regional amyloid burden and MEG metrics, a general linear model of the following form was used for each relevant neural response: MEG response ~ SUVr + age. Age was included in the null model for these analyses, as was the group for the linear regressions on cognitive function. Logistic and general linear regression models were performed using the *stats* package in *R* [54]. All MEG data preprocessing, coregistration, and sensor- and source-level analyses were performed in the Brain Electrical Source Analysis software suite (BESA Research v6.1 and BESA MRI v2.0). Cluster-based permutation testing on MEG sensor-array data was performed in BESA Statistics (v2.0). Plotting of model residuals used *ggplot2* [55].

## Results

**Fig. 2:**
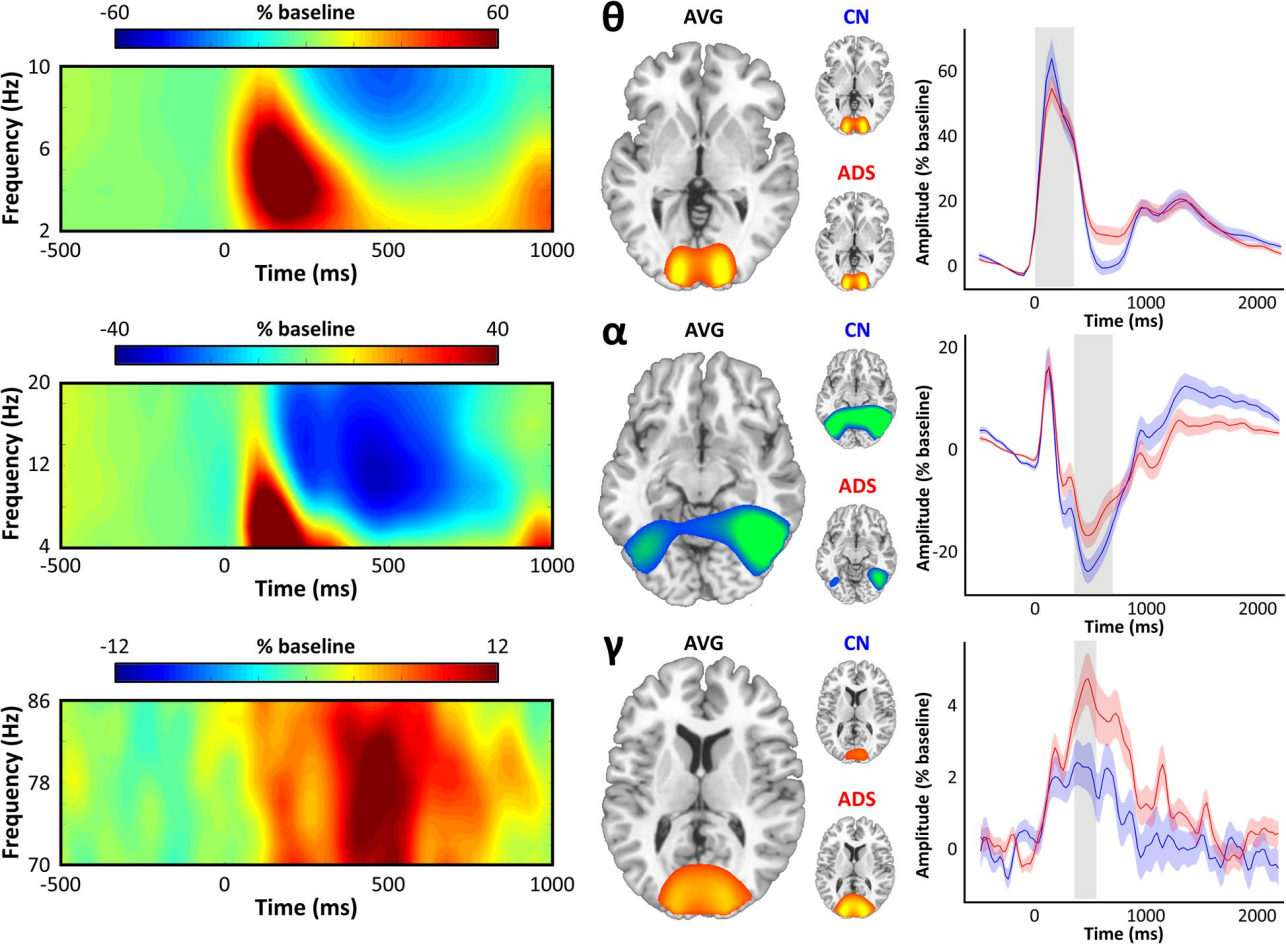
Multi-spectral occipital dynamics in patients on the Alzheimer’s disease spectrum. Spectrograms on the far left are representative sensors displaying the significant time–frequency responses identified in the sensor-level analysis, with percent change from baseline indicated on the scale bar above. Large brain maps in the middle represent occipital neural responses per each frequency band (top: theta; middle: alpha; bottom: gamma) that have been grand averaged over all participants. Smaller maps in the middle show these same responses averaged within each group (top: cognitively normal [CN] controls; bottom: Alzheimer’s disease spectrum [ADS]). Peak voxel virtual sensor time series per group are displayed on the right for these responses (averaged across hemispheres per group), with the gray shaded areas indicating time windows identified in the sensor-level analysis and imaged using a beamformer, and the colored shaded areas indicating ± 1 standard error of the mean

By design, performance on our visuospatial task was equal and near ceiling for both groups (Table 1; Fig. 1, bottom). As supported by stringent cluster-based permutation testing, as well as by numerous previous reports, we observed significant neural oscillatory responses to the visuospatial task stimuli in three temporally and spectrally defined windows (Fig. 2, left). These included an early synchronization (i.e., an increase from baseline levels of synchrony) in the theta band (0–350 ms; 3–6 Hz), followed by a strong de-synchronization (i.e., a decrease from baseline levels of synchrony) in the alpha band (350–700 ms; 8–14 Hz) and a synchronization in the gamma band (350–550 ms; 72–84 Hz). Imaging of these responses to the level of the cortex confirmed that they all originated from bilateral occipital regions, with the alpha response being more lateral than the medial theta and gamma responses (Fig. 2, middle). Inspection of the group-averaged response maps, as well as peak-voxel time series from these responses, subjectively suggested a pattern of differences in response amplitude, but not latency, between cognitively normal participants and patients on the AD spectrum (Fig. 2, right).

### Visuospatial neural oscillations differentiate patients on the AD spectrum from cognitively normal older adults

**Fig. 3:**
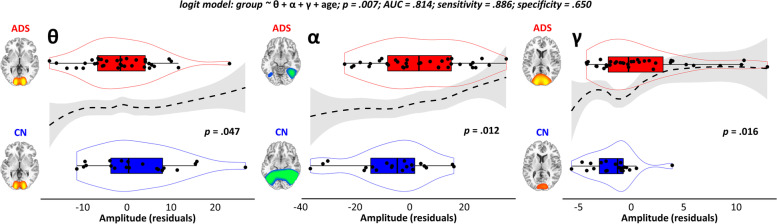
Classification of patients on the Alzheimer’s disease spectrum using multi-spectral occipital dynamics. The logistic regression model used to perform classification is displayed at the top, with the participant-level residuals and model fit for each frequency of neural response displayed in the graphs below (theta: left; alpha: middle; gamma: right). Box plots represent group residual means, first and third quartiles, and minima and maxima, and violin plots show the probability density. The black dashed line indicates the model fit across levels of the neural response amplitude (*x*-axis) and groups (*y*-axis). The significance of each response in contributing to the classification model (i.e., as a *p* value) is inlaid to the right, and the same group-averaged neural response maps are inlaid to the far left of each plot to aid in the interpretation

To examine the utility of these occipital neural dynamics for classifying patients on the AD spectrum, we next computed a logistic regression model with theta, alpha, and gamma frequency oscillatory responses as predictors, group as the binary dependent variable, and age as a predictor in the null model (Fig. 3, top). This model significantly predicted the differentiation of patients on the AD spectrum from cognitively normal older adults (*Χ*^2^(50) = 12.19, *p* = 0.007), and all three visuospatial oscillatory responses significantly contributed to the classification accuracy of the model. For the theta response in primary visual cortices and the alpha response in lateral occipital regions, patients on the AD spectrum exhibited reduced oscillatory amplitude (theta: *z* =  − 1.99, Wald = 3.95, odds ratio = 0.92, *p* = 0.047, Fig. 3, left; alpha: *z* = 2.53, Wald = 6.39, odds ratio = 1.07, *p* = 0.012, Fig. 3, middle), while the opposite was true for the gamma (*z* = 2.42, Wald = 5.84, odds ratio = 1.41, *p* = 0.016; Fig. 3, right) response in primary occipital cortices. Importantly, the alpha-band response was negative in sign (i.e., a desynchronization from baseline levels) so the larger (less negative) number indicates a reduced response in patients on the AD spectrum. Given the shape of the violin plots in Fig. 3, we computed the same model with an exclusionary threshold of ± 3 standard deviations from the mean, and the results were virtually unchanged. Thus, these effects were not due to the influence of outliers. We also tested for any potential prediction of these neural response amplitudes by regional amyloid-β burden (above and beyond the effects of age) using a general linear approach, but found no such relationship with the theta (*r* = 0.17, *p* = 0.330), alpha (*r* =  − 0.28, *p* = 0.110), or gamma (*r* =  − 0.16, *p* = 0.374) responses.

### Alpha and gamma oscillations predict cognitive status in patients on the AD spectrum

**Fig. 4:**
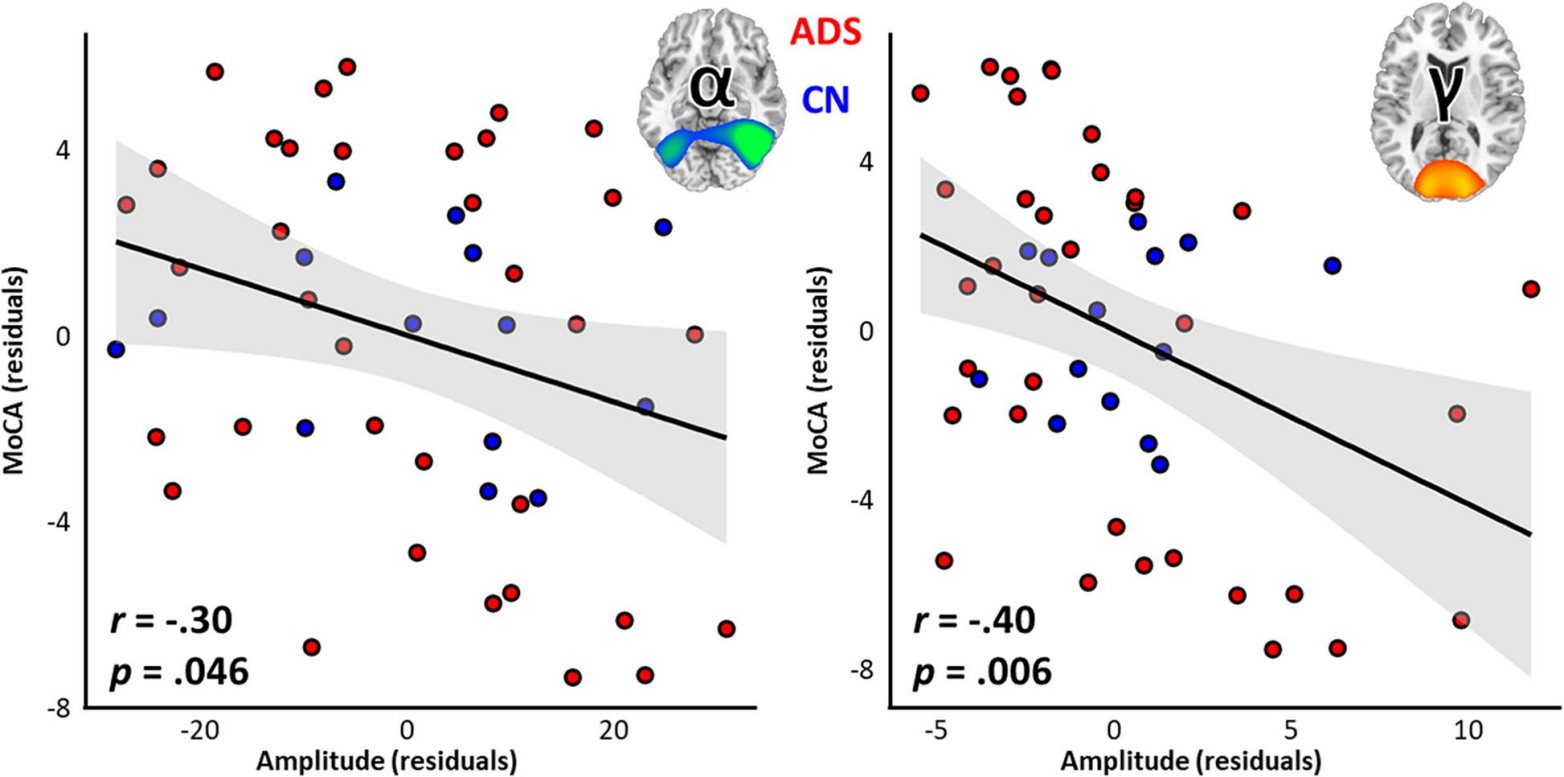
Alpha and gamma occipital dynamics predict global cognitive status in patients on the Alzheimer’s disease spectrum. Scatterplots represent significant relationships between neural response amplitude (*x*-axis; alpha: left; gamma: right) and global cognitive status as measured by the Montreal Cognitive Assessment (MoCA; *y*-axis). Individual participant data points for cognitively normal controls (CN) are shown in blue and for patients on the Alzheimer’s disease spectrum (ADS) in red, with lines-of-best-fit and corresponding confidence intervals overlaid

Finally, to determine the relevance of these visuospatial neural responses for predicting cognitive status in patients on the AD spectrum, we regressed the amplitude of the three responses on a test of general cognitive function (i.e., the MoCA). The full model was significant beyond the effects of group and age (Δ*F*(3,43) = 3.64; *p* = 0.020), suggesting that these dynamics are useful indicators of cognitive status in the early stages of AD. Post hoc investigation of the predictive capacity of each spectrally-defined neural response indicated that both the alpha (*r* =  − 0.30, *p* = 0.046) and gamma (*r* =  − 0.40, *p* = 0.006) responses, but not the theta response (*r* = 0.02, *p* = 0.902), significantly predicted general cognitive status (Fig. 4). For both the alpha and gamma band, the direction of this effect was intuitive, in that greater deviation from cognitively normal adults predicted worse cognitive status. A similar regression model on another widely used test of cognitive function (i.e., the MMSE) produced highly similar results (theta: *r* = 0.096, *p* = 0.503; alpha: *r* =  − 0.34, *p* = 0.013; gamma: *r* =  − 0.36, *p* = 0.010). Computing these models with outliers excluded (threshold of ± 3 SD from the mean) did not meaningfully change the results. Further, computing the same analysis in only the AD spectrum group produced largely similar results, with the exception of the alpha-MMSE and alpha-MoCA relationships becoming marginal (MoCA: *p* = 0.062; MMSE: *p* = 0.071), potentially reflecting a difference in the statistical power between the two approaches. Once again, computing these AD spectrum-only models with outliers excluded produced interpretationally identical results, with the notable exception of the alpha-MoCA relationship becoming significant (*r* =  − 0.40, *p* = 0.029).

## Discussion

New interest in rhythmic neuronal activity in patients with AD has spurred a huge number of basic and translational studies of visual processing; however, the dynamic patterns of such neural oscillatory activity in these patients are not well understood. In particular, although visuospatial processing is known to be impaired early in the course of the disease, the oscillatory neural responses known to support this process have not been investigated in patients with AD. In this study, we find that the multi-spectral occipital neuronal dynamics supporting visuospatial processing in healthy adults are uniquely impacted in patients on the AD spectrum. Specifically, patients exhibited blunted theta and alpha responses, alongside stronger gamma frequency synchronizations. These pathological deviations significantly differentiated patients on the AD spectrum from cognitively normal older adults and scaled with performance on common cognitive screening tools (i.e., MoCA and MMSE). These findings provide key new information, both for the field’s basic understanding of the cognitive neuropathology of AD, as well as for emerging clinical interventions aimed at frequency-targeted neurostimulation.

While all three neural oscillatory responses contributed significantly to the logistic regression classifier, the alpha and gamma oscillations appeared to be particularly important in this regard. In addition, these responses, and not the theta oscillations, significantly predicted cognitive status in these participants, indicating their potential utility for clinical relevance in the pathophysiology of AD-related cognitive decline. Interestingly, the group differences across these responses were bi-directional, such that gamma oscillations were enhanced and alpha responses were diminished in patients on the AD spectrum. In both cases, these deviations from cognitively normal adults appeared to index pathology; patients with weaker alpha responses and stronger gamma responses tended to exhibit the poorest cognitive status per total MoCA score. While alpha band neuronal aberrations in AD are well documented during the resting state (i.e., when no cognitive task is being performed; [28, 29, 56, 57]), much less is known regarding the impact of AD on task-induced alpha responses. Further, alpha rhythms are known to be particularly important for the spatially selective functional gating of incoming visual information [48, 58, 59], and this is the first evidence that the alpha oscillations supporting visuospatial processing are deviant in patients on the AD spectrum. In contrast to the alpha rhythm, much less evidence exists for differences in gamma-frequency oscillations between cognitively normal adults and patients with AD, and virtually no studies have investigated induced gamma responses in this population. The notable exception to this is a study wherein the authors examined gamma responses to a range of basic sensory stimuli and found a similar pattern of increased gamma response amplitude in patients on the AD spectrum [30]. Given the recent interest in the use of rhythmic gamma-frequency visual stimulation as a non-invasive treatment for AD, it seems highly relevant that we found such robust differences in this frequency. In terms of the underlying cognitive mechanisms, gamma oscillations are indicative of energetically expensive local processing [60] and are imperative for representing stimulus-specific information during visuospatial processing [61, 62]. Thus, our finding of stronger gamma responses that scale with cognitive decline in patients on the AD spectrum is suggestive of less efficient stimulus representation in these patients during the processing of their visuospatial environment.

The theta response did not contribute as robustly to the classification analysis as the alpha and gamma oscillations and exhibited no significant relationship with cognitive impairment. This was somewhat surprising, as the blunting of low-frequency oscillatory responses during visual processing is the most frequently reported effect in patients with AD [23–27, 63]. Generally, theta responses to visual stimuli are thought to support early alerting to salient stimuli within the visuospatial environment [14, 64, 65], and our findings suggest that such early visual processing is perhaps less essential in understanding the visuospatial deficits often experienced by patients with AD.

### Limitations

Limitations for this work must also be acknowledged. First, while we provide robust evidence for a dysfunctional pattern of rhythmic neuronal activity supporting visuospatial processing in these patients, very little is known regarding the impact of AD on the dynamics supporting higher-order attention and executive function. Previous research has established that multi-spectral neural activity in visual cortices is also essential for visuospatial attention, so in future studies, it will be important to probe whether the effects reported here transfer to and interfere with activity in fronto-parietal and other attention networks. Second, although we did find a significant relationship between visuospatial neural responses and general cognitive function (i.e., MoCA and MMSE scores) in patients on the AD spectrum, our more detailed neuropsychological battery did not include sub-tests specific to visuospatial processing. Thus, we were unable to connect these neural responses to visuospatial abilities measured outside of the scanner, which we would tentatively predict are being modeled by the shared variance with the MoCA and MMSE scores, since both of these tests incorporate visuospatial components. Third, although we found no significant relationship between regional amyloid burden and visuospatial oscillatory responses across patients, this does not necessarily indicate that no such relationship exists in AD. As amyloid-β is known to accumulate prior to clinically significant cognitive declines becoming apparent in these patients [66], it is possible that any such relationship would need to be tested in a much earlier, pre-clinical disease stage. Additionally, more nuanced modeling approaches that leverage within-participant spatial co-variability between MEG and PET have recently been successful in uncovering relationships between regional measures of proteinopathy and neural activity [67, 68]. While beyond the scope of this study, this type of modeling is an intuitive next step for this line of research. Finally, while our patient group spanned a wide range of clinical AD severity from early amnestic MCI to early dementia, we were unable to extend our analyses to people in the pre-clinical stages of the disease. This would be an essential next step to determine how early these occipital dynamics might be relevant for diagnostic purposes and would shed additional light on the relationship between these dynamics and cognitive declines in AD, as well as amyloid burden as mentioned above. Relatedly, although the current data provide novel information regarding the neural bases of visuospatial processing deficits in patients on the AD spectrum, it remains unclear whether these findings may also provide clinically meaningful gains in classification accuracy beyond what is possible with EEG resting-state protocols. Extensive electrophysiological work has supported the potential utility of such resting-state approaches [69] in confirming AD diagnoses, which makes this another appealing next line of inquiry.

## Conclusions

These findings shed new light on the neurophysiological underpinnings of visuospatial deficits in patients with AD. While theta, alpha, and gamma frequency neural responses to visuospatial stimuli differentiate patients on the AD spectrum from biomarker-negative older adults, alpha and gamma oscillations appear to be particularly important for tracking cognitive decline along this spectrum. Thus, occipital alpha and gamma frequency neural oscillations appear to be valid targets for ameliorating such deficits with emerging clinical interventions, as well as for tracking visuo-spatial decline in these patients.

## Data Availability

The datasets used and/or analyzed during the current study are available from the corresponding author on reasonable request.
